# Interventions to support children’s engagement in health-related decisions: a systematic review

**DOI:** 10.1186/1471-2431-14-109

**Published:** 2014-04-23

**Authors:** Bryan Feenstra, Laura Boland, Margaret L Lawson, Denise Harrison, Jennifer Kryworuchko, Michelle Leblanc, Dawn Stacey

**Affiliations:** 1 University of Ottawa, Faculty of Health Sciences, Ottawa, ON, Canada; 2 Children’s Hospital of Eastern Ontario Research Institute, Ottawa, ON, Canada; 3 Children’s Hospital of Eastern Ontario, Ottawa, ON, Canada; 4 University of Saskatchewan College of Nursing, Saskatoon, SK, Canada; 5 University of Ottawa, Health Sciences Library, Ottawa, ON, Canada; 6 Clinical Epidemiology Program, Ottawa Hospital Research Institute, Ottawa, ON, Canada

**Keywords:** Child, Adolescent, Decision making, Patient participation, Practice

## Abstract

**Background:**

Children often need support in health decision-making. The objective of this study was to review characteristics and effectiveness of interventions that support health decision-making of children.

**Methods:**

A systematic review. Electronic databases (PubMed, the Cochrane Library, Web of Science, Scopus, ProQuest Dissertations and Theses, CINAHL, PsycINFO, MEDLINE, and EMBASE) were searched from inception until March 2012. Two independent reviewers screened eligibility: a) intervention studies; b) involved supporting children (≤18 years) considering health-related decision(s); and c) measured decision quality or decision-making process outcomes. Data extraction and quality appraisal were conducted by one author and verified by another using a standardized data extraction form. Quality appraisal was based on the Cochrane Risk of Bias tool.

**Results:**

Of 4313 citations, 5 studies were eligible. Interventions focused on supporting decisions about risk behaviors (n = 3), psycho-educational services (n = 1), and end of life (n = 1). Two of 5 studies had statistically significant findings: i) compared to attention placebo, decision coaching alone increased values congruence between child and parent, and child satisfaction with decision-making process (lower risk of bias); ii) compared to no intervention, a workshop with weekly assignments increased overall decision-making quality (higher risk of bias).

**Conclusions:**

Few studies have focused on interventions to support children’s participation in decisions about their health. More research is needed to determine effective methods for supporting children’s health decision-making.

## Background

The perspective of the child is important when making decisions about his or her health [1-3]. When children are involved in decision-making, they experience decreased anxiety and an increased sense of value and control [3,4]. Their involvement is also thought to improve communication between children, parents and clinician(s); which is important for child/parent satisfaction and may also improve adherence with the chosen treatment [5,6]. The practice of including children in decision-making is also advocated by several prominent organizations. In 1989, the United Nations Convention on the Rights of a Child (UNCRC) provided grounds for a child’s right to be involved in decisions regarding his or her health [7]. The American Academy of Pediatrics Committee on Bioethics recommends that children should be included in decision-making to the greatest extent possible [8]. Children’s ability to make health decisions is influenced by multiple factors such as developmental stage, experience with the disease, and parental and health professional attitudes about the child’s capacity [9,10]. For example, in a recent study involving children with Type I diabetes making decisions with their parent and healthcare team, children as young as 8 years old were successfully recruited [11]. Therefore, the extent that children can participate in health decisions should depend on their ability and not their chronological age. As such, children’s competence should be assessed on an individual basis and in relation to the decision being made. Nonetheless, lack of competence should not be a reason to restrict children’s right to participate in decisions about their health [9].

Despite benefits and clear mandates for including children, studies show that children are not sufficiently involved and their preferences are not being elicited as often or consistently as they could be [9,10,12,13]. Furthermore, although most clinicians recognize the need to include children in decision-making, they have varying opinions about when and how to do so [14]. Factors such as the child’s age, length of illness, previous experiences, clinical condition, behavior, and ability to express oneself are often considered when deciding whether or not to include him or her [1,14,15]. As a result, children are often excluded, which may lead to fear, confusion, and anger on the part of the child [3,16].

A Cochrane review examined the effects of interventions that enhance general communication between health professionals and children with cancer [17]. Although some interventions demonstrated some benefit to children by improving knowledge, psychological support, and reintegration into school and social activities, the communication interventions in this review were not designed to address children’s decision-making needs. Another Cochrane review of interventions to support shared decision making in children with cancer had no studies that meet the inclusion criteria [18]. No other systematic review has specifically explored interventions tailored to support children in their health-related decision-making. The purpose of this systematic review was to explore the characteristics and effectiveness of interventions that support the decision-making needs of children who are actively considering a health-related decision.

## Methods

A systematic review was conducted using a protocol developed a priori based on the Cochrane Handbook for Systematic Reviews of Interventions [19]. Studies including children who were actively facing a health-related decision with or without their parent(s)/guardian(s) were considered for inclusion (see Table 1). Children were defined as individuals aged 18 years or younger [7]. Studies needed to evaluate an intervention that addressed an identified decision-making need of the child. Study designs considered were randomized controlled trials (RCTs), non-randomized controlled trials (non-randomized CT), interrupted time series (ITS), and controlled before-and-after (CBA) designs. Comparator groups could have been usual care or any alternative intervention. Study outcomes needed to address either the quality of the decision (e.g., knowledge, values-choice agreement) or the decision-making process (e.g., decisional conflict, satisfaction) for children. These outcomes are based on the International Patient Decision Aids Standards [20] and are consistent with systematic reviews of interventions to support adults and parents in making health decisions [21,22].

**Table 1:** Inclusion/exclusion criteria for article eligibility

	**Included**	**Excluded**
**Participants**	• Children (≤ 18 years) who are facing a health-related decision	• Children not treated as active participants in decision-making
	• Decisions about participation in health research	• Decisions not directly pertaining to their health or hypothetical decisions
**Interventions**	• Interventions to support children’s decision-making needs	• Interventions that support only the information needs of children
**Design**	• Randomized controlled trials	• Qualitative studies, descriptive studies, cohort studies
	• Non-randomized controlled trials	• Editorials, opinion articles
	• Interrupted time series	
	• Controlled before-and-after	
**Outcomes**	• Outcomes that affect the quality of the decision or the decision-making process for children/youth	• Studies that do not report at least one of the outcomes relating to the quality of the decision or the decision-making process
**Language**	• English or French	• Other languages
**Publication status**	• Published	• Unpublished studies
	• Peer-reviewed	• Non peer-reviewed

### Search strategy

The following electronic databases were searched: Evidence-Based Medicine Reviews (Ovid) (Cochrane Database of Systematic Reviews (Issue 2, 2012), Database of Abstracts of Reviews of Effects (1^st^ quarter 2012), Cochrane Central Controlled Trials Register (Issue 2, 2012)); MEDLINE (Ovid) (1966 to March 2012); MEDLINE (PubMed) (1945 to March 2012); CINAHL (via EBSCOhost) (1981 to March 2012); PsycINFO (1806 to March 2012); Web of Science (1898 to March 2012); Scopus (1960 to March 2012); ProQuest Dissertations and Theses (1861-March 2012); EMBASE (Ovid, 1974 to March 2012). The Agency for Healthcare Research and Quality (AHRQ) website (under Children’s Health) and Google Scholar were also searched informally using key words from the search strategy. Finally, reference lists of included articles and review articles were scanned.

The search strategy included a mix of subject headings and keywords related to the intervention (e.g., intervention, patient participation, social support, health communication), decision support techniques (e.g., decision-making-computer-assisted, decision trees), and decision-making (see Table 2). Some limits were applied relating to study types (e.g., clinical trials, or randomized controlled trials, or evaluation studies), language (English or French only), and participant types (must include child or adolescent).

**Table 2:** Search strategy used for Pubmed

**Group**	**Searched terms**
1	intervention* OR intervene* OR "Health Knowledge, Attitudes, Practice" [Mesh] OR "Social Support" [Mesh] OR "Family" [Mesh] OR "Patient Participation" [Mesh] OR "Health communication" [Mesh] OR "Health education" [Mesh] OR "Decision Support Techniques" [Mesh] OR "Decision Making, Computer-Assisted" [Mesh])
2	("Decision Making" [Mesh])
3	(Humans [Mesh])
4	(Clinical Trial [ptyp] OR Meta-Analysis [ptyp] OR Randomized Controlled Trial [ptyp] OR Review [ptyp] OR Classical Article [ptyp] OR Comparative Study [ptyp] OR Controlled Clinical Trial [ptyp] OR Evaluation Studies [ptyp] OR Historical Article [ptyp] OR Journal Article [ptyp] OR Multicenter Study [ptyp] OR Patient Education Handout [ptyp] OR Validation Studies [ptyp])
5	(English [lang] OR French [lang])
7	1 AND 2 AND 3 AND 4 AND 5
8	(infant [MeSH] OR child [MeSH] OR adolescent [MeSH])
9	7 AND 8

### Study selection

After removing duplicates, retrieved article citations were uploaded onto a web-based screening application designed by our research team’s information technologist. This program allows independent reviewers to evaluate study eligibility through a multi-stage screening process: titles, abstracts, and full-text. First, references identified by the search are loaded into the title screening application and randomly assigned to reviewers for initial screening. Excluded titles are assigned to another reviewer for screening. Reviewers do not know if they are screening first or second. All included citations then move to the second (abstracts) screening stage, using the same process. Title and abstract screenings were completed by BF and at least one other reviewer (LB, DS, ML, JK). Full-text versions were reviewed manually for final inclusion by BF and LB. Disagreements between reviewers were resolved by consensus or by consulting a third member (DS) of the review team.

### Data collection

Data extraction was conducted by BF and verified by a second review author (LB). The process was guided by a data extraction form based on one used in another systematic review of decision support interventions [22]. The data extraction sheet was piloted with a randomly selected study chosen for inclusion and necessary revisions to the form were made. Disagreements between review authors regarding data extraction were resolved by discussion.

The following information was extracted from each study (as per the data extraction sheet): a) characteristics of child participants (location, age, gender, ethnicity, diagnosis, and stage of illness), b) study methods (aims, design, allocation, recruitment, inclusion/exclusion criteria, informed consent, ethical approval, funding, and statistical methods), c) intervention(s) and control intervention(s) (enrollment and attrition of participants, type(s), co-interventions, content, mode of delivery, timing, frequency, duration, provider, training, and elements of decision support), d) outcomes (primary and secondary measures, definition(s), methods of follow-up, timing, validity of instruments used and adverse events), e) results (according to study type), and f) limitations and conclusions indicated by the original authors.

### Risk of bias assessment

The Risk of Bias tool from the Cochrane Handbook was used to assess RCTs [19]. Risk of Bias tables adapted using guidelines developed by the Cochrane Effective Practice and Organization of Care Review Group [23] were used to assess studies with non-randomized CT, ITS and CBA designs.

Quality assessment was completed independently by two reviewers (BF and a research assistant). Disagreement was resolved through discussion, and when unsuccessful, a third reviewer (DS) arbitrated. As suggested by the Cochrane Handbook, the following types of bias were assessed as “high risk”, “low risk”, or “unclear risk”: a) selection bias (random sequence generation and allocation concealment), b) performance bias (blinding of participants and personnel), c) detection bias (blinding of outcome assessment), d) attrition bias (incomplete outcome data), e) reporting bias (selective reporting), and f) other bias.

### Measures

The primary outcomes of interest for this systematic review were those that improved decision quality: knowledge regarding the decision and options, accuracy of perceptions regarding benefits and harms of treatment options, and agreement between values and chosen option. Secondary outcomes were those that improved the decision-making process: satisfaction with process, decisional conflict, participation in decision-making process, communication with health professional and parent(s)/guardian(s), and proportion undecided. Outcome results were presented as reported in studies.

### Data synthesis

The limited number of eligible studies and heterogeneity in interventions, study design and outcomes precluded the pooling of results for meta-analysis. A descriptive synthesis was therefore conducted. The synthesis of findings was structured using the following domains: characteristics of studies, interventions, and outcome measures; and impact of interventions. Studies with similar interventions were grouped together. The following intervention categories were used: a) decision coaching alone (coaching), b) coaching plus an educational aid, and c) education alone. Essential elements of decision support interventions were identified with criteria previously used to evaluate decision support technologies and general SDM interventions [22,24-26].

## Results

### Studies selected

**Figure 1:**
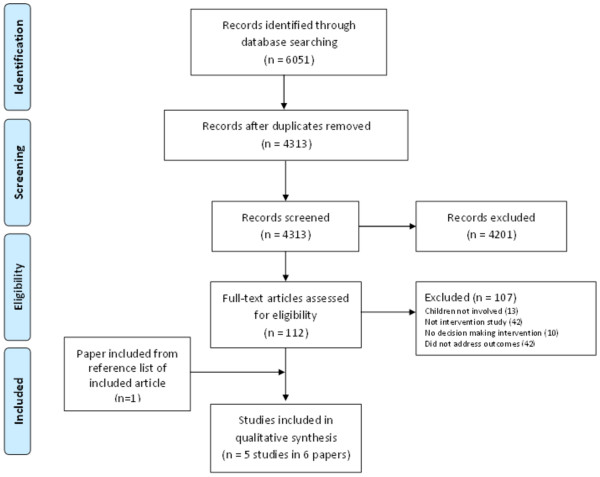
Literature flow diagram.

The search identified 6051 citations. After removing duplicates, 4313 original articles were screened (see Figure 1). Of these, 4201 citations were removed after title and abstract screening because they did not meet the inclusion criteria. The full text reports of 112 citations were retrieved and 107 citations were excluded. The results of 1 study were published in 2 papers; therefore, after retrieving the additional paper, this review included 5 studies published in 6 papers.

All 5 studies were conducted in the United States and published in English (see Table 3). Three studies published since 2008 were RCTs [27-29] and 2 studies published before 2000 were a non-randomized CT [30] and a CBA study [31]. Studies included a variety of decision types including participating in risk behaviors (n = 3), choosing a psycho-educational service to overcome learning problems (n = 1), and end of life planning (n = 1). Four studies were conducted in a clinical setting and 1 was conducted in a day camp.[31] Sample size of participants in included studies ranged from 38 to 819 (median 64). Three studies were conducted with children who had a chronic medical condition (asthma, HIV or cancer) [28,29,31,32] and 2 were conducted with children without any previous medical concerns [27,30]. Of the 5 included studies, 2 RCTs were rated as lower risk of bias, 1 RCT had an unclear risk of bias due to vague reporting, and 2 non-RCTs had higher risk of bias (see Table 3) [30,31].

**Table 3:** Characteristics of included studies (N = 5)

**Author (year)**	**Study design**	**Decision**	**Participants (n) and setting**	**Comparisons**	**Primary outcome(s)**	**Quality assessment**		
Rhee, 2008 [28]	RCT	Partaking in risk behaviors	41 children with asthma (20^a^ + 21^b^); 4 rural outpatient clinics and 1 high school	Coaching and computer based program v. attention placebo	Feasibility of the decision-making program	^d^: Low Risk	^g^: Low Risk	^j^: Low Risk
						^e^: Low Risk	^h^: Unclear	
						^f^: Low Risk	^i^: Unclear	
Lyon, 2009 [27,31]	RCT	End of life decision-making	40 children with HIV and their parents (21^a^ + 19^b^); 2 hospital outpatient clinics	Coaching v. attention placebo	Communication quality, congruence of treatment preferences, decisional conflict satisfaction	^d^: Low Risk	^gg^: Unclear	^j^: Low Risk
						^e^: Low Risk	^h^: Low Risk	
						^f^: Unclear	^ii^: Unclear	
Adams, 2009 [26]	RCT	Sun exposure v. sun protection	819 children (395^a^ + 424^b^); primary care physicians office	Coaching and computer program v. attention placebo	Sun protection behaviors, pros for protection, pros for exposure, decisional balance	^d^: Unclear	^g^: Unclear	^j^: Low Risk
						^e^: Unclear	^h^: Unclear	
						^f^: Low Risk	^i^: Unclear	
Hollen, 1999 [30]	CBA	Partaking in risk behaviors	64 cancer-surviving children (21^a^ + 43^c^); campground	Workshop and weekly assignments v. no intervention	Decision-making, risk motivation, risk behaviors	^d^: High Risk	^g^: Unclear	^j^: Low Risk
						^e^: High Risk	^h^: Unclear	
						^f^: High Risk	^i^: Low Risk	
Adelman, 1990 [29]	Non-randomized CT	Psycho-educational decision-making	85 families (32^a^ + 20^b^ + 33^c^); university clinic	Pre-conference coaching v. no intervention v. attention placebo	Child participation	^d^: High Risk	^g^: High Risk	^j^: Low Risk
						^e^: High Risk	^h^: Unclear	
						^f^: High Risk	^i^: High Risk	

^a^Intervention group.

^b^Placebo group.

^c^No intervention group.

^d^Random sequence generation.

^e^Allocation concealment.

^f^Blinding of participants AND personnel.

^g^Blinding of outcome assessment.

^h^Incomplete outcome data.

^i^Selective reporting.

^j^Other sources of bias.

### Decision support interventions

Decision support interventions were decision coaching [27-30,32] or an educational workshop [31] and were accompanied by computer programs, workbook exercises, telephone follow-ups, and information packages. Control groups received no intervention [31] and/or an attention placebo such as a computer program, coaching, or information package on another topic not related to the decision (see Table 4) [27-30,32]. Of the 12 essential elements of decision support interventions, [33] 1 study addressed 11 elements, [28,32] 3 studies addressed 6 elements, [27,29,31] and 1 study addressed 4 elements (see Table 5) [30].

**Table 4:** Characteristics of Decision Support Interventions (N = 5)

**Study (year)**	**Group**	**Decision support program**	**Administered by:**	**Intervention and timeline**	**Intervention duration**
Rhee, 2008 [28]	Decision support	Coaching guided by risk behavior fact sheet. Computer-based decision-making module.	Healthcare Provider	Main intervention plus CD-ROM intervention booster at 2 and 4 mo. post-intervention	Coaching = 10 min, Computer = 60 min,
		Intervention boosters: computer based decision-making module, workbook, and substance prevention computer program.			2 mo. Booster = 90 min
					4 mo. booster = 30 min
	Control	Sham computer program of comparable length featuring study skills.	Participant directed	Computer program only	Comparable to the intervention program minus the booster
		No booster.			
Lyon, 2009 [27,31]	Decision support	Three semi-structured interviews: 1. *Lyon Family Centered Advance Care Planning Survey*, 2. The *Respecting Choices* patient centered-ACP interview, 3. *Five Wishes* legal directive.	Trained Facilitator	Three sessions, 1 week apart	180- 270 min. (for three sessions)
	Control	Three sessions re: 1. non-medical developmental history, 2. safety information, 3. career planning.	Trained Facilitator	Three sessions, frequency not specified	Comparable to the intervention
Adams, 2009 [26]	Decision support	Brief coaching, interactive computer sessions, telephone assessments, printed tailored feedback, a brief printed manual, mailed tip sheets, and samples of sunscreen.	Healthcare provider/	Main intervention at baseline and 12 months	Coaching session = 2 to 3 min.
			Participant directed	At 3, 6, 15, and 18 mo. children phoned for the expert system assessments	Sun Smart System = 20 min
					Follow up assessments = not specified
	Control	Computer program with monthly stage-matched telephone calls, printed manual and mail at 24 mo. Information related to physical activity, sedentary behavior, total fat intake, and servings per day of fruits and vegetables.	Trained Facilitator	Stage matched to intervention group	Not specified
Hollen, 1999 [30]	Decision support	Camp workshop integrating survivorship, quality decision-making skills, children risk behaviors, and social support from peers and health professionals. Follow up workbook exercises with audio-tape.	Trained Facilitator	Workshop plus 4 weekly assignments	Workshop = 1 day.
					Weekly assignments = not specified
	Control	No intervention.		Not specified	Not specified
Adelman, 1990 [29]	Decision support	Pre-conference coaching encouraging and facilitating child’s participation in the conference.	Trained Facilitator	Main intervention only	5 to 15 min
	Control	1. Attention placebo-expanded neutral explanation of the conference process.	Trained Facilitator	Control 1: Explanation only	Control 1: Not specified
		2. No-intervention.		Control 2: Not specified	Control 2: Not specified

**Table 5:** Elements of the decision support interventions (N = 5)

**Intervention description**	**Rhee, 2008 [**[28]**]**	**Lyon, 2009 [**[27]**,**[31]**]**	**Adams, 2009 [**[26]**]**	**Hollen, 1999 [**[30]**]**	**Adelman, 1990 [**[29]**]**
**Type of intervention**					
Coaching alone		✓			✓
Coaching and educational aid	✓		✓		
Education alone				✓	
**Elements of decision support**					
Decision defined/explained	✓	✓		✓	✓
Assess/discuss patient’s decision-making needs		✓	✓		
Options (including alternatives) presented	✓	✓	✓	✓	
Benefits of options discussed	✓	✓	✓	✓	
Risks of options discussed	✓	✓		✓	
Understanding assessed/clarified		✓	✓		
Values/preferences discussed	✓	✓	✓	✓	
Build skills in deliberation, communication, and accessing support		✓			✓
Ability/self-efficacy to enact plan discussed					✓
Decision made or explicitly deferred		✓			
Facilitate progress in decision-making	✓	✓	✓	✓	✓
Follow-up arranged		✓			
**Total Elements**	**6**	**11**	**6**	**6**	**4**

✓= decision support element present.

### Outcome measures

Four studies had 1 or more primary outcomes related to decision quality [27,28,30-32] and 2 studies had 1 or more outcomes related to the decision-making process [28,30,32] (see Table 6). Quality of decision-making was measured in 2 studies using the Decision-Making Quality Scale [34]. Satisfaction with the decision-making process was measured in 2 studies using unpublished scales. Other decision-making outcomes included: agreement between values and chosen option, congruence of treatment preferences between child and parent, participation in decision-making process, decisional conflict, and communication [27,28,30,32]. Outcomes that were not related to decision quality or the decision-making process were sun protection behaviors, [27] motivational readiness and future motivation, [30] risk motivation and actual risk behaviors (e.g., smoking, alcohol use, and illicit drug use), [29,31] and feasibility of a decision-making program [29].

**Table 6:** Summary of outcomes examined and statistical significance (N = 5)

**Comparisons**	**Coaching alone v. attention placebo/No intervention**		**Coaching plus aid v. attention placebo/No intervention**		**Education alone v. attention placebo/No intervention**
**Study**	**Lyon 2009 [**[27]**,**[31]**]**	**Adelman 1990 [**[29]**]**	**Adams 2009 [**[26]**]**	**Rhee 2008 [**[28]**]**	**Hollen 1999 [**[30]**]**
**Decision quality**					
Overall quality of the decision-making process				No statistically significant difference	Statistically significant at 1 *P* = 0.02 and 12 months *P* = 0.001, but not 6 months post-intervention
Congruence for values and chosen option			No statistically significant difference		
Child–parent congruence for treatment option preference	Statistically significant difference on 1 of 3 scenarios				
**Decision-making process**					
Satisfaction	Statistically significant for 2 of 3 intervention components. (*P* = 0.001)	No statistically significant difference			
Participation		No statistically significant difference			
Decisional conflict	Statistically significant informed sub-score (*P* = 0.001)				
Communication	No statistically significant difference				

### Effectiveness of interventions

#### Decision coaching alone versus attention placebo/no intervention (n = 2 studies)

Two of the 5 studies compared coaching with an attention placebo or no intervention (Table 6) [28,30,32]. In the Lyon study, [28] decision coaching consisted of trained facilitators who elicited and stimulated conversation about patients’ views and opinions about their disease. In the Adelman study, decision coaches encouraged children to participate, and facilitated a discussion about participation strategies. Then the decision coach and child rehearsed participation strategies [30].

For decision quality outcomes, 1 study [28] reported improved values congruence between parent and child for 1 of the 3 scenarios tested. There were no statistically significant differences for the low survival and functional impact scenario (as it related to HIV end of life decisions); however, improved parent–child congruence was found for the cognitive impairment scenario (69%; CI 0.45-0.90 vs. 11%; CI 0.05-0.25, congruence) [28].

For decision-making process outcomes, Lyon and colleagues [32] found that children were more satisfied with the decision-making process (*P* = 0.001) while another study [30] reported no difference. One study [28] found no difference in decisional conflict scores (except for a sub-score relating to feeling informed (*P* = 0.001), and no difference in the quality of child-decision coach communication. One study [30] found no difference in the child’s level of participation in health decision-making. Original reports on the decision-making process outcomes did not include descriptive statistics of outcome measure scores.

#### Coaching plus educational aid versus attention placebo/no intervention (n = 2 studies)

For decision quality outcomes, coaching combined with a co-intervention had no effect on agreement between participants’ values and their chosen behavior when compared to an attention placebo in 1 study [27]. There was no difference in overall quality of decision-making when compared to an attention placebo in the other study [29].

#### Education alone versus attention placebo/no intervention (n = 1 study)

In one study, an educational workshop with weekly assignments increased decision-making quality in one of three scenarios presented. Compared to the control group, the intervention decision-making quality scores improved in the cognitive impairment scenario at 1 (mean difference of 0.34 vs. 1.62, *P* = 0.02) and 12 months (−0.38 vs. 1.79, *P* = 0.001), but not 6 months post-intervention (mean difference of 0.23 vs. 1.05, *P* = 0.10) [31]. Higher scores indicate better decision quality.

## Discussion

This systematic review was designed to evaluate the characteristics and effectiveness of interventions that support children in health decision-making. Although interventions to support decision-making in the adult setting have been well tested, [22] the evaluation of formal interventions supporting pediatric health related decision-making is lacking. Our systematic review identified only 5 studies of which 4 evaluated decision coaching with or without a co-intervention aid (e.g., computer programs, workbook exercises, information packages), 1 evaluated an educational workshop, and none evaluated patient decision aids with decision coaching. Interestingly however, 3 of the 5 studies included in this review were published within the last 5 years, which may indicate a growing interest in evaluating interventions to support children’s decision-making.

Two studies had statistically significant findings: coaching alone increased agreement between parent and child values (i.e., values congruence) between child and parent as well as child satisfaction with the decision-making process (1 RCT), and education alone increased overall decision-making quality (1 CBA study). Three studies found no difference in decision-making quality, satisfaction with the decision-making process, and child participation in decision-making (2 RCTs, 1 non-randomized CT). We could not comment on the clinical significance of the findings because either the scales used to measure the outcome lacked psychometric properties or the effect size of significant results were not provided in the original article.

Coaching was part of the decision support interventions in 4 of 5 studies. The study by Lyon and colleagues, [28,32] which was one of the higher quality studies that met most elements of decision support, used a coaching alone intervention for end-of-life decision-making. It found increased values congruence between child and parent, and increased child satisfaction with the decision-making process compared to controls. These findings are consistent with a systematic review of decision coaching interventions that found adults were more satisfied when decision coaching was used alone or in conjunction with patient decision aids compared to usual care or a patient decision aid alone [35]. Interestingly, the positive study included in our review coached both parents and children together, [28,32] whereas other studies coached children only [27,29,30]. Decision coaching with both children and parents may be important for shared decision making within pediatrics as it can prepare all stakeholders who have an impact on the outcome and implementation of the decision [36,37].

Coaching was also provided together with educational resources such as computer programs, workbook exercises, and information packages [27,29]. Adams and colleagues [26] demonstrated that participants could establish clear values, and found correspondence between those values and chosen behavior; however they did not find a difference based on intervention. Rhee and colleagues [29] also found no difference based on intervention. These educational interventions appear to be similar to patient decision aids, which help prepare individuals to make a decision with their health professional [22]. However, education alone may not fully support decision-making as it does not address the patient’s contextual and social influences [21,22]. A systematic review of the decision-making needs of parents concluded that parents require not only timely, reliable, and current information but also support for the preference-sensitive nature of many decisions [21]. In contrast to the simple patient education resources evaluated in the studies included in this systematic review, patient decision aids better support SDM by also making explicit that a decision needs to be made, providing values clarification, and guiding patients through a stepped approach to thinking about the decision [22].

A review evaluating patient decision aids with adults found they increase knowledge, accuracy of risk perceptions, and the consistency of decisions with patient values [22]. Patient decision aids also lower decisional conflict (related to feeling uniformed and having unclear values), decrease indecision, and increase participation in decision-making. Since these interventions are successful with adult populations, it is possible that educational aids that account for the social and values-dependent nature of decision-making may be an effective intervention with children. However, similar to decision coaching, little research has been conducted regarding their use with either children and/or their parents.

There are several limitations that should be considered when interpreting the results of this systematic review. First, on an individual study level, there were few studies from which to draw firm conclusions. Furthermore, included studies lacked homogeneity with regards to patient context, interventions used, outcomes, and outcome measures; thereby precluding the pooling of results for meta-analysis. The overall quality of included studies ranged from low to high, with only 2 studies adequately meeting the risk of bias criteria. Another limitation was the lack of detail provided about interventions, potentially preventing an accurate assessment of the elements of decision support.

On a review level, although a thorough and systematic approach was used to search the literature with two independent reviewers screening citations, it is possible that relevant studies were missed. This review did not search trial registries and grey literature that may have contained studies that could contribute understanding to this topic. This review may also have been limited by restricting the search to English and French articles [38].

## Conclusions

Five studies, of variable quality, evaluated interventions to support children in making health decisions, with most of these studies published within the last five years. Despite increasing interest in supporting children’s participation in health decision making, this systematic review affirms the need for further research examining targeted interventions to support the involvement of children in SDM. Future studies evaluating interventions to support children’s decision-making should use rigorous designs such as randomized control trials or cluster randomized control trials, using outcome measures with evaluated psychometric properties, and clear and detailed reporting of decision support interventions and results.

## Abbreviations

RCT: Randomized controlled trial; SDM: Shared decision-making.

## Competing interests

The authors declare that they have no competing interests.

## Authors’ contributions

BF was involved in conception and study design, collection and analysis of data, wrote the first draft of the manuscript and edited and revised subsequent drafts. MLB, ML, DH and DS participated in study design, collection of data, and edited and revised the article for important intellectual content. JK and ML participated in data collection and edited and revised the article for important intellectual content. All authors approved the final manuscript as submitted.

## Pre-publication history

The pre-publication history for this paper can be accessed here:

http://www.biomedcentral.com/1471-2431/14/109/prepub
